# Exploring the Potential of the Corpus Callosum Area as a Predictive Marker for Impaired Information Processing in Multiple Sclerosis

**DOI:** 10.3390/jcm12216948

**Published:** 2023-11-06

**Authors:** Shun Akaike, Tomoko Okamoto, Ryoji Kurosawa, Nozomi Onodera, Youwei Lin, Wakiro Sato, Takashi Yamamura, Yuji Takahashi

**Affiliations:** 1 Department of Neurology, National Center of Neurology and Psychiatry, Tokyo 187-8551, Japan; akaike.shun.a6@elms.hokudai.ac.jp (S.A.); yutakahashi@ncnp.go.jp (Y.T.); 2 Department of Immunology, National Center of Neurology and Psychiatry, Tokyo 187-8551, Japan

**Keywords:** multiple sclerosis, cognitive impairment, magnetic resonance imaging

## Abstract

Early cognitive impairment (CI) detection is crucial in multiple sclerosis (MS). However, it can progress silently regardless of relapse activity and reach an advanced stage. We aimed to determine whether the corpus callosum area (CCA) is a sensitive and feasible marker for CI in MS compared to other neuroimaging markers. We assessed cognitive function in 77 MS patients using the Symbol Digit Modalities Test, Paced Auditory Serial Additions Task, Wechsler Adult Intelligence Scale-IV, and Wechsler Memory Scale-Revised. The neuroimaging markers included manually measured CCA, two diffusion tensor imaging markers, and nine volumetric measurements. Apart from volumes of the hippocampus and cerebellum, ten markers showed a significant correlation with all neuropsychological tests and significant differences between the groups. The normalized CCA demonstrated a moderate-to-strong correlation with all neuropsychological tests and successfully differentiated between the CI and cognitively normal groups with 80% sensitivity and 83% specificity. The marker had a large area under the curve and a high Youden index (0.82 and 0.63, respectively) and comparability with established cognitive markers. Therefore, the normalized CCA may serve as a reliable marker for CI in MS and can be easily implemented in clinical practice, providing a supportive diagnostic tool for CI in MS.

## 1. Introduction

Early detection of cognitive impairment (CI) is crucial in multiple sclerosis (MS) as it may lead to a decreased quality of life in young patients [1]. Recent studies have indicated that an appropriate selection of disease-modifying drugs can help treat these conditions. Identifying CI early allows for prompt treatment, improving outcomes in MS patients [2,3,4]. However, early identification of CI is challenging due to its insidious progression independent of relapse activity, often reaching an advanced stage before patients and neurologists become aware of it [5,6,7]. In the early stages, patients with MS may only exhibit slow information processing while learning, memory, and verbal skills are relatively preserved. This presents diagnostic challenges for CI [8,9]. Neuropsychological tests such as the Symbol Digit Modalities Test (SDMT) and the Paced Auditory Serial Additions Task (PASAT) are crucial in identifying impaired information processing speed, which is the primary cognitive dysfunction in MS [9]. However, these tests have certain limitations: first, they should not be administered frequently due to practice effects associated with repeated use [10]; second, cognitive dysfunction may be overlooked in a single-point assessment if patients have higher cognitive abilities before onset; third, the available scores may not be ideal for detecting subtle changes.

Numerous studies have demonstrated the utility of neuroimaging markers in providing supporting evidence of impaired information processing in MS [11,12,13,14,15,16,17,18]. For example, some neuropsychological tests have shown a correlation with the volume of the basal ganglia such as the thalamus [11,12], putamen [12], globus pallidus [12], caudate [12], and the brain parenchyma [13], lesions of MS [14], cerebellum [15], hippocampus [16], diffusion tensor measurements of the cingulum [17], and corpus callosum [18]. However, these markers are not widely used in routine clinical practice due to the requirement for specialized and time-consuming techniques for brain analysis, such as volumetric or diffusion tensor analysis. In contrast to these markers, atrophy of the corpus callosum may prove useful as it does not require specialized skills [18,19,20,21]. The corpus callosum index (CCI) is commonly used to assess the corpus callosum size without volumetry and has shown a correlation with cognitive function in MS [18,20,21]. The corpus callosum area (CCA) is another indicator used to quantify atrophy, although it is less commonly employed. CCA can be normalized to the head size [19], and a previous study demonstrated that CCA was more sensitive than CCI in predicting CI in MS [22]. Nevertheless, there is still a paucity of extensive studies examining the utility of CCA in predicting cognitive function in MS patients.

This study aimed to determine whether CCA is a sensitive and feasible marker for CI in MS compared to other previously established neuroimaging markers.

## 2. Methods

### 2.1. Selection of Patients

A total of 77 patients with MS were recruited cross-sectionally from the Department of Neurology at the National Center of Neurology and Psychiatry, Tokyo, Japan, between February 2021 and September 2022. All patients fulfilled the 2017 McDonald’s criteria. For patients with MS who underwent physical examination, the Expanded Disability Status Scale (EDSS), several neuropsychological tests (SDMT, PASAT, Wechsler Adult Intelligence Scale-IV (WAIS-IV), and Wechsler Memory Scale-Revised (WMS-R)), and the magnetic resonance imaging (MRI) of the head at the hospital were included in the study. The presence or absence of callosal disconnection syndrome was determined through physical examination. Patients with the following characteristics were excluded: those with neuromyelitis optica spectrum disorders (NMOSDs) or myelin oligodendrocyte glycoprotein antibody-associated disorders, those who had experienced recent relapses in the past three months, and those with a history of central nervous system disorders other than MS. This study adhered to the principles outlined in the Declaration of Helsinki. Due to the study’s cross-sectional nature and all procedures being performed as part of routine clinical care, ethical approval and the requirement for informed consent were waived by the local Ethics Committee of the National Center of Neurology and Psychiatry.

### 2.2. Neuropsychological Tests

To evaluate cognitive function, we utilized the SDMT, PASAT, WAIS-IV, and WMS-R. In the PASAT, single digits were presented at intervals of two seconds (PASAT 2) and one second (PASAT 1). The WAIS-IV comprises four index scores that represent significant components of intelligence: the verbal comprehension index (VCI), perceptual reasoning index (PRI), working memory index (WMI), and processing speed index (PSI). The WMS-R includes two measurements: general memory and delay recall. The SDMT and PASAT scores were converted into z-scores based on age-specific normative data from the Clinical Assessment for Attention, developed and standardized by the Japan Society for Higher Brain Dysfunction [23]. The SDMT, PASAT, and WAIS-PSI are used to assess information processing speed, which is the most affected cognitive domain in MS. Patients with a z-score ≤ −2 on the SDMT were categorized into the cognitive impairment (CI) group, while patients with a z-score > −2 on the SDMT were classified as cognitive normal (CN) [19]. Group categorization was based on the SDMT score, as it is one of the key assessments for evaluating cognitive function in MS [8,24].

### 2.3. MRI Data Acquisition

All patients underwent whole-brain MRI using a 3T system (Philips Medical Systems, Best, the Netherlands, or Siemens, Munich, Germany) with a 32-channel head coil. Patients were randomly assigned to one of the two MRI scans in clinical settings. The following sequences were acquired for all patients: a sagittal 3D T1-weighted magnetization-prepared rapid gradient-echo sequence, a sagittal 3D fluid-attenuated inversion recovery sequence, and diffusion tensor images (DTIs). A sagittal 3D T1-weighted magnetization-prepared rapid gradient-echo sequence was acquired with the following parameters previously reported [25]: echo time (TE)/repetition time (TR) = 7.18/3.46 or 1800/2.26 ms, field of view = 261 × 261 or 250 × 250 mm, matrix size = 384 × 384 or 320 × 288, number of excitations = 1, slice thickness = 0.6 or 0.8 mm, and 300 or 224 continuous transverse slices. Additionally, a sagittal 3D fluid-attenuated inversion recovery sequence was obtained with the following parameters: TE/inversion time (TI)/TR = 4700/1600/290 or 5000/1800/413 ms, field of view = 260 × 234 or 250 × 250 mm, matrix size = 512 × 460 or 261 × 261, number of excitations = 2 or 1, slice thickness = 0.55 or 1.0 mm, and 340 or 176 continuous transverse slices. DTIs were acquired using previously reported parameters [25]. The DTIs were acquired in the axial plane with the following parameters: TR/TE = 5760/62 or 8300/73 ms, matrix size = 80 × 80 or 76 × 76, field of view = 240 × 240 mm, 60 or 55 continuous transverse slices, and slice thickness of 3 mm with no interslice gap. To improve the signal-to-noise ratio, the acquisition was performed twice. Diffusion was measured along 15 or 12 noncollinear directions using a diffusion-weighted factor of 1000 s/mm², and one image was obtained without any diffusion gradient.

### 2.4. CCA Measurement

**Figure 1:**
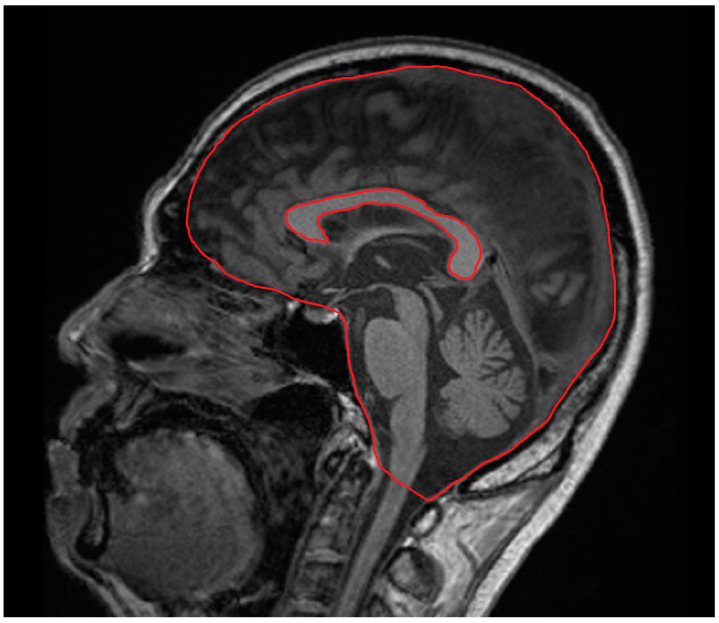
Techniques for manually measuring the corpus callosum area and intracranial skull surface area on midline T1-weighted magnetic resonance imaging sequence.

CCA was manually measured on a sagittal midline T1-weighted sequence using a picture archiving and communication system (PACS) by a neurologist (SA). The intracranial skull surface area was manually measured on the same image (Figure 1). The CCA was normalized to the intracranial skull surface area to account for head size. To assess intrarater reliability for normalized CCA (nCCA), we calculated the intraclass correlation coefficient based on ratings obtained during a second session three months later. Interrater reliability for nCCA was evaluated by comparing the ratings of other authors (TO and RK). All MRI assessments were randomized, with examiners blinded to the clinical assessments and ratings of the other examiners.

### 2.5. Volumetric Neuroimaging Markers

Using volumetry, we obtained nine neuroimaging markers: volumes of the brain parenchyma, cortex, putamen, globus pallidus, caudate, thalamus, hippocampus, cerebellum, and lesions of MS. The FreeSurfer software version 7.2.0 (https://surfer.nmr.mgh.harvard.edu/ (accessed on 5 November 2023)) was used to obtain the neuroimaging markers, except for lesion volumes. The sum of volumes in the bilateral hemispheres was measured in the putamen, globus pallidus, caudate, thalamus, and hippocampus. Total lesion volumes were measured using statistical parametric mapping (SPM) software version 12 (https://www.fil.ion.ucl.ac.uk/spm/ (accessed on 5 November 2023)) and the lesion segmentation toolbox (LST) version 3.0.0 (https://www.applied-statistics.de/lst.html (accessed on 5 November 2023)), which is specialized in MS lesion segmentation. Additionally, we measured the estimated total cranial volume (eTCV) with FreeSurfer. All volumetric markers were normalized to eTCV to account for head size. FreeSurfer and SPM enable automated segmentation of brain structures, and all segmentations were visually examined and, if necessary, manually edited by the author (SA).

### 2.6. DTI Neuroimaging Marker

Using DTIs, we analyzed the fractional anisotropy (FA) of the body of the corpus callosum and cingulate gyrus. The mean FA of the bilateral measurements was calculated for the cingulate gyrus. DTI was performed using MRtrix version 3 (https://www.mrtrix.org/ (accessed on 5 November 2023)). All DTIs underwent denoising, automatic removal of the Gibbs ringing artifact, and preprocessing to correct for eddy-current and echo-planar imaging-induced distortions. The data were then bias-field-corrected using the “-ants” option. Masks were automatically generated based on the bias-field-corrected images. The diffusion tensor model was fitted to each voxel, and an FA map was generated using the corrected masks on the bias field-corrected images. For localization of the body of the corpus callosum, we utilized the ‘JHU ICBM-DTI-81 White-Matter Labels’ atlas tools in the FMRIB Software Library (FSL version 6.0) (https://fsl.fmrib.ox.ac.uk/fsl/fslwiki/FSL (accessed on 5 November 2023)). We measured the FA of the corpus callosum’s body and the mean FA of the bilateral cingulate gyrus. All images with overlays were visually evaluated, and if the overlay was unsuccessful, they were manually edited by the author (SA).

### 2.7. Statistical Analysis

All statistical analyses were conducted using Easy R (EZR) software version 1.54 (Saitama Medical Center, Jichi Medical University, Saitama, Japan), which is a graphical user interface for R version 4.0.3 (R Foundation for Statistical Computing, Vienna, Austria) [26]. Logistic regression analyses were performed to assess differences between the CN and CI groups, while the Mann–Whitney U test was used to compare demographic characteristics. Spearman’s rank correlation coefficient was used to evaluate correlations between neuroimaging markers, neuropsychological tests, and EDSS. These analyses were adjusted for age, sex, years of education, and MRI scanner type. Additionally, Spearman’s rank correlation coefficient was calculated for individual neuropsychological tests. Correlation coefficients (ρ) ranging from 0.2 to 0.4, 0.4 to 0.6, 0.6 to 0.8, and 0.8 to 1.0 were considered weak, moderate, strong, and very strong, respectively [19]. Intrarater and interrater reliabilities were examined using the intraclass correlation coefficient (ICC). ICC values below 0.40, between 0.40 and 0.75, and above 0.75 were interpreted as poor, fair-to-good, and excellent based on statistical convention [19]. The significance threshold for all statistical tests was set at *p* < 0.05 due to the exploratory nature of the study and limitations in statistical power.

Receiver operating characteristic (ROC) curves were generated for the results of SDMT, incorporating the 12 MRI markers, with the determination of the cutoff value, sensitivity, specificity, and areas under the curve (AUC). Youden indices, which represent the highest sum of sensitivity and specificity, were calculated for all parameters. Additionally, an AUC analysis was performed on a multivariate set of all MRI markers and other explanatory variables (age, sex, education years, and MRI scanner type), employing the stepwise Akaike information criterion method to identify the optimized predictive model for CI in MS.

## 3. Results

The characteristics of the CN and CI groups are presented in Table 1. The groups had significant differences in age and MS subtype (*p* < 0.05). Callosal disconnection syndrome did not occur in any patients.

The neuropsychological test scores and neuroimaging marker measurements are summarized in Table 2.

**Figure 2:**
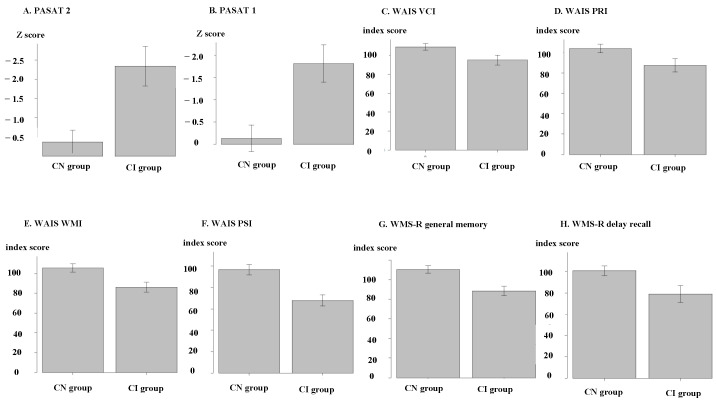
Neuropsychological results in cognitively normal and impaired groups. Bar heights represent the mean, and error bars represent individual neuropsychological tests’ 95% confidence interval. (**A**) PASAT 2 z-score, (**B**) PASAT 1 z-score, (**C**) WAIS VCI, (**D**) WAIS PRI, (**E**) WAIS WMI, (**F**) WAIS PSI, (**G**) WMS-R general memory, and (**H**) WMS-R delayed recall. CN: cognitive normal; CI: cognitive impairment; PASAT: Paced Auditory Serial Additions Task; WAIS: Wechsler Adult Intelligence Scale; VCI: verbal comprehension index; PRI: perceptual reasoning index; WMI: working memory index; PSI: processing speed index; WMS-R: Wechsler Memory Scale-Revised.

Among the 12 neuroimaging markers, 10 showed significant differences between the two groups, excluding the normalized cerebellum and hippocampus volumes. The neuropsychological results of the two groups are presented in Figure 2. The correlation analyses between neuroimaging markers and neuropsychological test scores are shown in Table 3. The WAIS VCI score did not significantly correlate with any neuropsychological test. Among the 12 neuroimaging markers, 10 showed significant correlations with all neuropsychological tests except for WAIS VCI: nCCA, FA of the body of the corpus callosum and cingulate gyrus, normalized volumes of the brain parenchyma, cortex, thalamus, putamen, globus pallidus, caudate, and normalized lesion volume. The nCCA demonstrated moderate to strong correlations with all neuropsychological tests except for the WAIS VCI. The EDSS score showed weak or no correlation with any of the MRI markers, and these correlations were weaker than those observed with neuropsychological tests.

**Figure 3:**
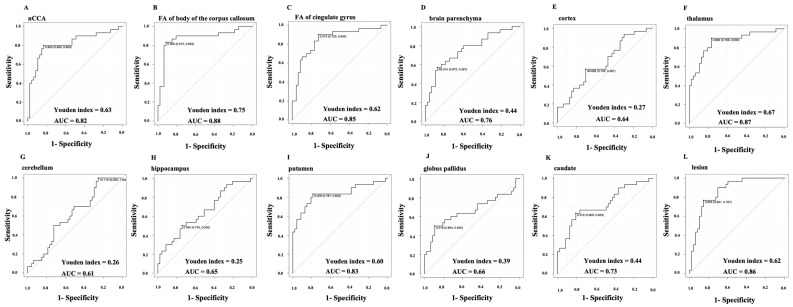
Receiver operating characteristic (ROC) curves for the MRI markers. ROC curves are shown for the 12 MRI markers and the values of the Youden index and area under the curve (AUC). The point on each ROC curve represents the cutoff value, sensitivity (right), and specificity (left). (**A**) nCCA, (**B**) FA of the corpus callosum, (**C**) FA of the cingulate gyrus, (**D**) normalized brain parenchyma, (**E**) normalized cortex volume, (**F**) normalized thalamus volume, (**G**) normalized cerebellum volume, (**H**) normalized hippocampal volume, (**I**) normalized putamen volume, (**J**) normalized globus pallidus volume, (**K**) normalized caudate volume, and (**L**) normalized lesion volume. nCCA, normalized corpus callosum area; MS, multiple sclerosis; MRI, magnetic resonance imaging.

The ROC curves for individual neuroimaging markers are presented in Figure 3. The sensitivity, specificity, cutoff, and AUC are summarized in Table 4. In nCCA, the sensitivity and specificity were 80% and 83%, respectively, and the AUC and Youden index was relatively high (0.82 and 0.63, respectively). Multivariate analysis of multiple MRI markers revealed that the best predictive model included the variables, age, FA of the corpus callosum, and brain parenchyma, resulting in an AUC of 0.895.

The interrater and intrarater ICC for nCCA were excellent (0.91 and 0.92, respectively; *p* < 0.01).

## 4. Discussion

In the present study, nCCA was significantly correlated with CI in patients with MS. This study has shown that the marker offers a diagnostic value for CI in MS, and the interrater and intrarater ICC suggested the robustness of this marker. The Youden index of nCCA was the third highest, following the FA of the body of the corpus callosum and normalized volume of the thalamus, while the AUC of nCCA was the sixth largest among markers. To the best of our knowledge, this is the first study to comprehensively compare previous neuroimaging markers to emphasize the usefulness of nCCA.

The two markers appeared superior to nCCA in predicting CI in MS, but nCCA has several advantages. First, nCCA can be quickly measured without these special skills or tools. Second, problems associated with volumetry and/or DTI analysis can be avoided in the measurement of nCCA. The accuracy of these techniques can be affected by abnormalities in the brain, including numerous MS plaques or strong atrophy of the brain structures [27]. Diffusion tensor analysis has the same shortcomings, and its accuracy is also affected by newly formed MS lesions, which are associated with increased FA [28]. Third, nCCA may be less influenced by MRI scanners and measurement conditions. In contrast, volumetry and diffusion tensor analysis are strongly impacted by technical factors, including the type and parameters of the MRI scanner, the software used for analysis, and the analysis methods employed [29].

We calculated individual AUC values for each marker. This approach aligns with the primary aim of our study, which was to evaluate the utility of nCCA in comparison to other MRI markers. In fact, combining individual markers did not yield significant improvements in predictive models. Multivariate analysis produced the highest AUC value of 0.895, while analysis using a single MRI marker, specifically FA of the corpus callosum, showed the highest AUC value of 0.88. This marginal improvement is because all the markers are reflective of the same underlying cause, namely, white matter damage resulting from MS plaques. Potential underlying mechanisms are discussed below.

In this study, we selected SDMT, PASAT, WAIS-IV, and WMS-R as neuropsychological assessments instead of neuropsychological tests specifically designed for CI in MS, such as the brief repeatable battery of neuropsychological tests (BRB-N) [30] or the brief international cognitive assessment for multiple sclerosis (BICAMS) [31]. The primary reason for this choice is that BRB-N is not standardized in Japan, and although BICAMS is standardized in Japan [32], it is not readily accessible to all neurologists and is routinely used in clinical settings. As a result, we opted to combine multiple tests ourselves. SDMT and PASAT play a crucial in identifying information processing impairment, which is the primary cognitive disturbance in MS. WMS-R, comprehensive memory scales available in Japan, allow us to assess memory deficits, a common concern in MS, as assessed by BRB-N and BICAMS. WAIS-IV provides a measure of general IQ, which is important because a decrease in IQ can complicate the assessment of other cognitive domains.

Our data suggest that impaired information processing is a fundamental cognitive problem in MS, as shown in a previous report [9]. Figure 2 suggests that most of the patients in the CN group showed normal results in all tests. This suggests that cognitive dysfunction is confined to impaired information processing, resulting in secondary dysfunction in reasoning, working memory, memory, and other cognitive domains; scores of these neuropsychological tests, such as WMS-R, WAIS-PRI, and WAIS-WMI, were decreased because of impaired information processing. WAIS-VCI was relatively preserved both in CN and CI groups because verbal ability was not strongly correlated with information processing.

The mechanisms of corpus callosum atrophy in MS have not yet been elucidated, but several potential explanations exist for the reduction in nCCA. The corpus callosum has rich reciprocal connectivity with the brain and may be particularly susceptible to secondary degeneration due to MS lesions in the cerebral white matter. A previous report showed that fibers passing through the corpus callosum were injured in MS [33]. Plaques in the cerebral white matter and corpus callosum may play an important role in the atrophy of the corpus callosum. We also considered that cognitive dysfunction is not directly related to lesions in the corpus callosum. In our study, none of the patients presented with callosal disconnection syndrome. Accumulating MS lesions can cause disconnection of multiple cognitively relevant tracts, resulting in cognitive dysfunction and atrophy of anatomical structures with rich reciprocal connectivity with the brain.

Our study has several limitations. First, this was a single-center study in Japan, and a selection bias might have influenced the results. However, Western-type MS in Asia is not fundamentally different from typical MS in Western countries [34]. Second, the extent to which these markers are sensitive to the progression of MS was not revealed because of the study’s cross-sectional nature. Third, the number of patients with primary progressive MS was limited. Fourth, we did not include healthy subjects in this study due to hospital regulations that prohibit the use of MRI scans on individuals without a clinical indication. The absence of healthy controls is associated with reduced reliability in determining cutoff values, as we cannot provide reference values for MRI markers. Therefore, we did not aim to establish an optimal cutoff value for MRI markers in this study. However, including healthy controls is not an absolute necessity for our primary objective, which is to evaluate the utility of nCCA compared with other established MRI markers. To overcome these limitations, we will perform a prospective study by following up on these patients and recruiting new patients with MS and healthy subjects.

## 5. Conclusions

In conclusion, we revealed that nCCA might be a reliable and easy-to-use biomarker of CI in MS. nCCA can be easily translated into clinical practice because volumetric or diffusion tensor analysis is not required, providing a supporting diagnostic tool for CI in MS.

## Figures and Tables

**Table 1:** Demographic data of patients with multiple sclerosis.

	Overall	Cognitive Normal Group	Cognitive Impairment Group	
	*n* = 77	*n* = 47	*n* = 30	*p*-value
	mean (SD)	mean (SD)	mean (SD)	
Age, year	47.94 (9.67)	49.85 (9.16)	44.93 (9.83)	<0.05
Sex, *N*, f/m	54/23	36/11	18/12	0.56
Education year, year	13.48 (2.17)	13.60 (2.25)	13.30 (2.05)	0.82
EDSS, median	4.50 (3)	4.50 (4.00)	4.50 (2.38)	0.21
Disease duration, year	13.13 (8.94)	12.64 (10.36)	13.90 (6.19)	0.43
Subtype of MS, *N*, RR/SP/PP	44/26/7	33/10/4	11/16/3	<0.05
Callosal disconnection syndrome, N	0	0	0	

*N*: number; f/m: female/male; EDSS: Expanded Disability Status Scale; MS: multiple sclerosis; RR: relapsing-remitting; SP: secondary progressive; PP: primary progressive.

**Table 2:** Scores of neuropsychological tests and measurements of neuroimaging markers.

	Overall	Cognitive Normal Group	Cognitive Impairment Group	
	*n* = 77	*n* = 47	*n* = 30	*p*-value
	mean (SD)	mean (SD)	mean (SD)	
SDMT z-score	−1.7 (1.6)	−0.6 (0.9)	−3.4 (1.0)	<0.001
PASAT 2 z-score	−1.2 (1.5)	−0.5 (1.1)	−2.2 (1.4)	<0.001
PASAT 1 z-score	−0.8 (1.2)	−0.2 (0.9)	−1.8 (1.0)	<0.001
WAIS VCI	102.1 (14.3)	107.4 (12.0)	93.7 (13.8)	<0.001
WAIS PRI	96.6 (16.9)	102.9 (14.1)	86.8 (16.4)	<0.001
WAIS WMI	97.3 (15.4)	104.2 (13.5)	86.6 (11.9)	<0.001
WAIS PSI	84.9 (19.8)	96.1 (14.3)	67.3 (13.3)	<0.001
WMS-R general memory	95.3 (18.5)	104.2 (13.2)	81.5 (17.3)	<0.001
WMS-R delay recall	91.8 (20.0)	100.2 (15.0)	78.6 (19.9)	<0.001
nCCA	2.9 (0.9)	3.3 (0.7)	2.3 (0.8)	<0.001
corpus callosum, FA	0.48 (0.08)	0.52 (0.05)	0.42 (0.07)	<0.001
cingulate gyrus, FA	0.39 (0.06)	0.42 (0.05)	0.35 (0.06)	<0.001
brain parenchyma ^a^	70.1 (9.1)	73.2 (8.3)	65.3 (8.2)	<0.05
cortex ^a^	41.1 (5.0)	42.2 (5.2)	39.4 (4.4)	<0.05
thalamus ^a^	0.87 (0.17)	0.95 (0.15)	0.75 (0.13)	<0.001
cerebellum ^a^	9.1 (1.2)	9.3 (1.4)	8.7 (0.9)	0.14
hippocampus ^a^	0.53 (0.09)	0.55 (0.09)	0.50 (0.08)	0.55
putamen ^a^	0.55 (0.13)	0.60 (0.10)	0.48 (0.13)	<0.001
globus pallidus ^a^	0.25 (0.04)	0.26 (0.04)	0.24 (0.05)	<0.05
caudate ^a^	0.41 (0.08)	0.43 (0.07)	0.37 (0.07)	<0.05
lesion ^a^	1.0 (1.6)	0.54 (0.81)	1.8 (2.2)	<0.001

Logistic regression analysis was performed after adjusting for age, sex, education duration, and MRI scanner type. nCCA: normalized corpus callosum area; FA: fractional anisotropy; MRI: magnetic resonance imaging; SDMT: Symbol Digit Modalities Test; PASAT: Paced Auditory Serial Additions Task; WAIS: Wechsler Adult Intelligence Scale; VCI: verbal comprehension index; PRI, perceptual reasoning index; WMI: working memory index; PSI: processing speed index; WMS-R: Wechsler Memory Scale-Revised. ^a^ normalized volume of the anatomical structures.

**Table 3:** Results of correlation analyses between neuroimaging markers and scores of neuropsychological tests as well as EDSS.

	SDMT	PASAT 2	PASAT 1	WAIS VCI	WAIS PRI	WAIS WMI	WAIS PSI	WMS-R General Memory	WMS-R Delay Recall	EDSS
nCCA	0.60 *	0.40 *	0.54 *	0.10	0.46 *	0.49 *	0.60 *	0.61 *	0.63 *	−0.26 *
corpus callosum, FA	0.67 *	0.49 *	0.59 *	0.13	0.52 *	0.55 *	0.67 *	0.63 *	0.66 *	−0.24 *
cingulate gyrus, FA	0.65 *	0.52 *	0.56 *	0.13	0.52 *	0.58 *	0.60 *	0.59 *	0.65 *	−0.21
brain parenchyma ^a^	0.43 *	0.36 *	0.49 *	0.17	0.39 *	0.44 *	0.53 *	0.46 *	0.44 *	−0.20
cortex ^a^	0.26 *	0.24 *	0.33 *	0.15	0.27 *	0.33 *	0.38 *	0.26 *	0.26 *	−0.22
thalamus ^a^	0.55 *	0.42 *	0.58 *	0.23 *	0.38 *	0.46 *	0.59 *	0.56 *	0.59 *	−0.22
cerebellum ^a^	0.19	0.10	0.25 *	0.03	0.17	0.24 *	0.27 *	0.24 *	0.16	−0.14
hippocampus ^a^	0.25 *	0.18	0.28	0.08	0.19	0.22	0.31	0.34 *	0.36 *	−0.14
putamen ^a^	0.55 *	0.48 *	0.58 *	0.22	0.47 *	0.57 *	0.56 *	0.57 *	0.62 *	−0.14
globus pallidus ^a^	0.23 *	0.20	0.36 *	0.12	0.18	0.29 *	0.29 *	0.31 *	0.28 *	−0.10
caudate ^a^	0.36 *	0.39 *	0.44 *	0.07	0.35 *	0.36 *	0.39 *	0.49 *	0.45 *	−0.12
lesion ^a^	−0.62 *	−0.40 *	−0.45 *	−0.13	−0.47 *	−0.39 *	−0.61 *	−0.66 *	−0.70 *	0.30 *

All comparisons are Spearman ρ coefficients after adjusting for age, sex, education duration, and types of MRI scanners. * *p* < 0.05. ^a^ normalized volume of the anatomical structures. EDSS: Expanded Disability Status Scale; nCCA: normalized corpus callosum area; FA: fractional anisotropy; PASAT: Paced Auditory Serial Additions Task; WAIS, Wechsler Adult Intelligence Scale; VCI: verbal comprehension index; PRI: perceptual reasoning index; WMI: working memory index; PSI: processing speed index; WMS-R: Wechsler Memory Scale-Revised.

**Table 4:** Sensitivity, specificity, cutoff, AUC, and Youden index of individual neuroimaging markers.

	Sensitivity (%)	Specificity (%)	Cutoff	AUC	Youden Index
nCCA	80	83	2.86	0.82	0.63
corpus callosum, FA	83	92	0.47	0.88	0.75
cingulate gyrus, FA	90	72	0.41	0.85	0.62
brain parenchyma ^a^	57	87	65.5	0.76	0.44
cortex ^a^	57	70	39.7	0.64	0.27
thalamus ^a^	90	77	0.86	0.87	0.67
cerebellum ^a^	100	26	10.1	0.61	0.26
hippocampus ^a^	50	75	0.50	0.65	0.25
putamen ^a^	83	79	0.53	0.83	0.60
globus pallidus ^a^	50	89	0.22	0.66	0.39
caudate ^a^	63	81	0.37	0.73	0.44
lesion ^a^	77	85	0.91	0.86	0.62

^a^ normalized volume of the anatomical structures. nCCA: normalized corpus callosum area; FA: fractional anisotropy.

## Data Availability

Data are available on reasonable request.
